# Targeted mutagenesis of *SlGAD3* generates very high levels of GABA in commercial tomato cultivars

**DOI:** 10.1007/s42994-025-00249-w

**Published:** 2025-09-22

**Authors:** Lei Zhu, Shiyang Zhang, Qingfeng Niu, Yansha Li, Xiaomu Niu, Pengcheng Wang, Jian-Kang Zhu, Zhaobo Lang

**Affiliations:** 1 Institute of Advanced Biotechnology, Institute of Homeostatic Medicine, School of Medicine, Southern University of Science and Technology, Shenzhen, 518055 China; 2 Shandong Shunfeng Biotechnology Co. Ltd., Jinan, 250000 China; 3 National Engineering Laboratory of Crop Stress Resistance Breeding, School of Life Sciences, Anhui Agricultural University, Hefei, 230036 China

**Keywords:** Gamma-aminobutyric acid, Tomato, Gene editing, Glutamate decarboxylase

## Abstract

GABA, a non-proteinogenic amino acid with anti-hypertensive properties, holds health-beneficial potential when enriched in crops. Previous studies have established that targeted disruption of the calmodulin-binding domain (CaMBD) of the tomato glutamate decarboxylase 3 (SlGAD3) enhances GABA biosynthesis. In this study, we used CRISPR/Cas9-mediated gene editing to precisely modify the CaMBD coding sequence of *SlGAD3* in three elite tomato varieties (SFT1, SFT2, and SFT3). Under our experimental conditions, targeted editing of *SlGAD3* led to substantial accumulation of GABA in all three varieties without compromising key agronomic traits such as fruit size and number. Although flowering was delayed in *SFT2* and *SFT3* mutants, *SFT1* mutants had higher GABA levels but also maintained a wild-type flowering time. This result highlights the critical importance of selecting specific varieties, such as SFT1, to minimize pleiotropic effects. By identifying varieties that can accumulate high levels of GABA without major reductions in growth and yield potential, this work bridges a critical gap between plant metabolic-engineering research and practical applications in commercial crop-improvement programs.

**Supplementary Information:**

The online version contains supplementary material available at 10.1007/s42994-025-00249-w.

Dear Editor,

Gamma-aminobutyric acid (GABA) is a ubiquitous non-proteinogenic amino acid found in bacteria, animals, and plants. Multiple empirical studies have shown that exogenous GABA administration can reduce blood pressure in hypertensive subjects (Takahashi et al. 1955). GABA-enriched foods have also shown anti-hypertensive effects and could be a useful preventative measure against hypertension (Inoue et al. 2003), suggesting that rice, tomatoes, and other products with enhanced GABA levels could potentially have health-beneficial applications.

Tomatoes are an important vegetable crop with a notable ability to concentrate high levels of GABA in their fruit (Jia et al. 2024). GABA content changes dramatically during tomato fruit development, accumulating before the breaker stage and declining thereafter (Akihiro et al. 2008). The GABA shunt diverts metabolic flux away from the tricarboxylic acid cycle by omitting the sequential oxidative decarboxylation of α-ketoglutarate to succinate. This bypass is achieved through the sequential catalytic action of three enzymes: glutamate decarboxylase (GAD), GABA aminotransferase, and succinate semialdehyde dehydrogenase (Bouché and Fromm 2004).

Plant GAD is a typical pyridoxal-5′-phosphate-dependent decarboxylase that assembles into hexameric quaternary structures (Gut et al. 2009) and exhibits a distinctive molecular signature not observed in its animal and bacterial counterparts. This uniqueness is attributed to the presence of a conserved C-terminal extension, ranging from 30 to 50 amino acid residues, which constitutes the calmodulin-binding domain (CaMBD) (Yap et al. 2003). This domain is crucial for modulating enzyme activity and integrating GAD into plant-specific signaling networks. Research on transgenic plants has shown that deletion of the CaMBD increases GABA levels, suggesting that the CaMBD may help activate GAD with Ca^2+^/CaM under stress. In the absence of Ca^2+^/CaM, the CaMBD acts as an auto-inhibitory domain (Baum et al. 1996). New types of plant GADs have recently been identified in various crops. Tomato SlGAD2 and SlGAD3 have been shown to significantly enhance GABA biosynthesis in tomato plants when the CaMBD is disrupted (Takayama et al. 2015). A recent study demonstrated that CRISPR/Cas9 mutagenesis of the C-terminal region of SlGAD3 substantially increased GABA accumulation from less than 100 μg/g in wild-type (WT) fruits of Micro-Tom to as high as 1,250 μg/g in T1 mutant fruits (Nonaka et al. 2017). Although the dwarf tomato Micro-Tom is a useful model for laboratory research on some aspects of tomato biology owing to its compact size and rapid life cycle, it is not a commercial cultivar. In addition, its accelerated secondary metabolite biosynthesis leads to premature accumulation of defense compounds. These inherent constraints highlight the role of Micro-Tom as a specialized research tool rather than a general model for commercial tomato varieties (Altpeter et al. 2016). Fruits of commercial tomato varieties often have relatively high levels of GABA, ranging from several hundred to over one thousand μg/g. Here, we investigated whether editing of *SlGAD3* could generate very high levels of GABA in elite tomato varieties.

The last 24 amino acids in the C-terminal region constitute the CaMBD of SlGAD3 (Nonaka et al. 2017). We designed a gRNA and used CRISPR/Cas9-mediated genome editing to target the C-terminal domain of SlGAD3 in three elite varieties, SFT1, SFT2, and SFT3 (Fig. 1A). We performed Sanger sequencing of the targeted gene in genomic DNA from T1 transgenic plants to identify plants with homozygous mutations. For each variety, we isolated three different *SlGAD3* mutant lines (Fig. 1B). Alignment of DNA and amino acid sequences from the WT and the mutants revealed that all mutations led to premature stop codons before the CaMBD in SlGAD3 (Fig. 1B).

**Fig. 1:**
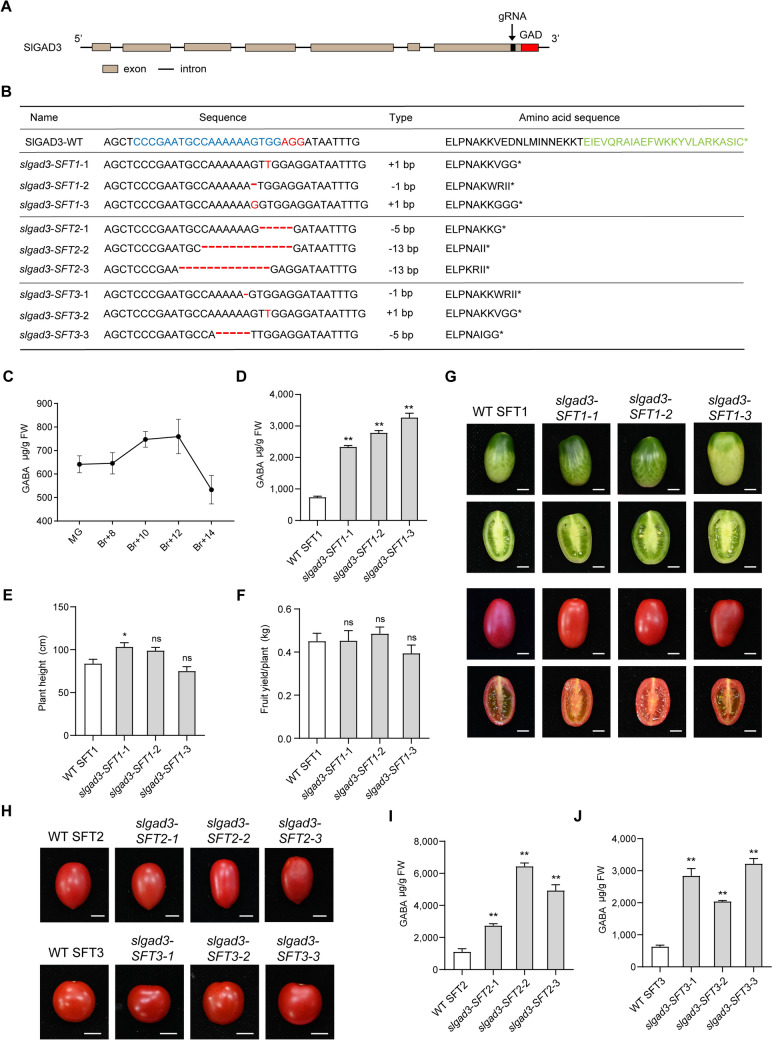
Effect of *SlGAD3* mutation on the GABA content of tomato fruit. **A** *SlGAD3* gene structure, highlighting exons and introns, and the gRNA target site for gene editing. **B** Gene and amino acid sequences of the wild-type (WT) *SlGAD3* gene and its mutants in three tomato cultivars (SFT1, SFT2, and SFT3). Mutations are indicated in red, and the amino acid sequences of CaMBD are shown in green. **C** GABA content of tomato fruits at five developmental stages: MG (mature green), Br + 8, Br + 10, Br + 12, and Br + 14. **D** GABA content of WT SFT1 and SFT1 mutant fruits at the Br + 12 stage. **E** Height of 12-week-old WT SFT1 plants and *slgad3-SFT1*-1, *slgad3-SFT1*-2, and *slgad3-SFT1-3* mutant lines (*n* = 9). **F** Total fruit yield per plant of WT SFT1 and *slgad3-SFT1*-1, *slgad3-SFT1*-2 and *slgad3-SFT1-3* mutant lines. Fruits were measured at the Br stages (*n* = 9). **G** Photographs of fruits from WT SFT1 and three SFT1 mutant lines at the MG and Br + 12 stages. Scale bars, 1 cm. **H** Photographs of fruits from WT SFT2, WT SFT3 and corresponding mutant lines at the Br + 12 stage. Scale bars, 1 cm. **I-J** GABA content of WT SFT2 and SFT3 and corresponding mutant fruits at the Br + 12 stage. Data are shown as mean ± SEM (*n* = 6). *P < 0.05; **P < *0.01* (Student’s *t-*test)

The GABA content of WT tomato fruits fluctuated across five developmental stages. Fruit at the mature green stage contained baseline levels of GABA. As the fruit progressed through the breaker stages, GABA content increased significantly, peaking at Br + 12. This peak was followed by a slight decline at Br + 14, suggesting that GABA accumulation is closely tied to specific developmental transitions during tomato fruit ripening (Fig. 1C).

We next compared the GABA contents of fruits from WT SFT1, *slgad3-SFT1*-1, *slgad3-SFT1*-2, and *slgad3-SFT1-3* at the Br + 12 stage and observed that GABA contents were substantially higher in the mutant lines than in the WT (Fig. 1D). Specifically, GABA contents were approximately 3.2 to 4.4 times higher in mutant fruits than in WT SFT1 fruits, reaching as high as 3,269 μg/g fresh weight (FW) in fruits of *slgad3-SFT1-3* (Fig. 1D). There were no significant differences in plant height, fruit yield per plant, or flowering time between the *slgad3-SFT1* mutants and the WT (Fig. 1E, F and S5). Photographs of fruits and plants of WT SFT1 and three *slgad3-SFT1* mutant lines at the mature green and Br + 12 stages are shown in Figs. 1G and S1. Together, these results show that SlGAD3 mutations increased GABA levels (Fig. 1D), but had no substantial effects on other agronomic traits in an elite tomato variety, highlighting the specific role of SlGAD3 in GABA metabolism.

To determine whether *SlGAD3* mutation also affected the GABA content of other elite varieties, we measured the GABA content of fruits from SFT2, SFT3, and their corresponding mutant lines at the Br + 12 stage. GABA content was substantially higher in *slgad3-SFT2* and *slgad3-SFT3* mutant fruits than in their corresponding WT fruits, consistent with our observations in SFT1 (Fig. 1I, J). Like those of SFT1, GABA contents of SFT2 and SFT3 were relatively high, reaching nearly 1000 μg/g in SFT2 fruits (Fig. 1D, I, J). Nonetheless, GABA content increased to over 6000 μg/g in some *slgad3-SFT2* mutant fruits (Fig. 1I). Photographs of fruits from WT SFT2, WT SFT3, and their corresponding mutants at the Br + 12 stage are shown in Fig. 1H.

Our study, thus, demonstrated that mutation of SlGAD3 to remove the CaMBD significantly enhanced GABA accumulation in three commercially relevant elite tomato cultivars. Crucially, the *SFT1* mutant lines accumulated high levels of GABA without compromising key agronomic traits, namely flowering time, plant height, and fruit yield. Although *SFT2* and *SFT3* mutant lines exhibited delayed flowering (Fig. S5), they showed no significant reductions in plant height (Figs. S2–S4) or fruit size (Fig. 1H) compared with their respective WTs. The exceptionally high GABA levels in these edited lines, particularly the *SFT1* mutant lines that showed minimal pleiotropic effects, position these tomatoes as promising candidates for the development of functional foods with potential health benefits. In contrast to experiments performed with Micro-Tom, our research bridges the gap between laboratory innovation and commercial application, providing valuable information for the development of genetically improved crops with enhanced GABA contents and potential health benefits.

## Materials and methods

### Plant materials and growth conditions

The *slgad3* mutants were generated by CRISPR/Cas9 gene editing using an sgRNA sequence (TCCTCCAATGGCATTCG) designed to target tomato *SlGAD3* (*Solyc01g00500*). A CRISPR/Cas9 vector was constructed and introduced into *Agrobacterium tumefaciens* EHA105 by chemical transformation. All constructs were confirmed by sequencing and then transformed into three tomato cultivars (SFT1, SFT2, and SFT3) by *Agrobacterium*-mediated transformation. T0-generation transgenic plants were obtained and self- pollinated to segregate the desired mutations from the CRISPR-Cas9 transgene locus. The T1 progeny were characterized by PCR to identify transgene-free individuals that were homozygous for the target mutation. All plants were grown in a growth room at 25 °C with a 16-h light/8-h dark photoperiod.

### Phenotypic analysis of WT and mutant tomato plants

Plant height, defined as the vertical distance from the base of the cotyledons to the apical meristem, was measured on 12-week-old WT and mutant plants. All ripe fruits were collected from each plant to evaluate fruit yield. *P* values were calculated using Student’s *t-*test (*P < 0.05; **P < 0.01).

### Measurement of GABA content

GABA was extracted from tomato fruits at the Br + 12 stage by homogenizing 10 mg of freeze-dried tissue in 1 mL ultrapure water and ultrasonicating for 30 min. After centrifugation at 12,000 × *g* and 4 °C for 15 min, the supernatants were filtered through 0.22-μm organic membranes (Merck). GABA levels were quantified by high-performance liquid chromatography–tandem mass spectrometry (UHPLC system, Vanquish Horizon; MS system, Q-Exactive; Thermo Fisher Scientific) using a BEH C18 column (2.1 × 100 mm, 1.7 μm) and a gradient of 0.1% formic acid in water (A) and acetonitrile (B) at a flow rate of 0.3 mL/min and a temperature of 40 °C. Analyses were performed at the core facility department of Shandong Shunfeng Biotechnology Co., Ltd.

## Supplementary Information

Below is the link to the electronic supplementary material.

Supplementary file1 (DOCX 3086 KB)

## Data Availability

The authors confirm that the data supporting the findings of this study are available within the article and its supplementary materials.
