# Supplementary material for: Targeted mutagenesis of SlGAD3 generates very high levels of GABA in commercial tomato cultivars
Source: aBIOTECH. 2025 Sep 22;6(4):693–7. doi: 10.1007/s42994-025-00249-w (PMC12647399; doi:10.1007/s42994-025-00249-w)
Supplement: Supplementary file 1 — Supplementary file1 (DOCX 3086 KB) [file 42994_2025_249_MOESM1_ESM.docx]

**
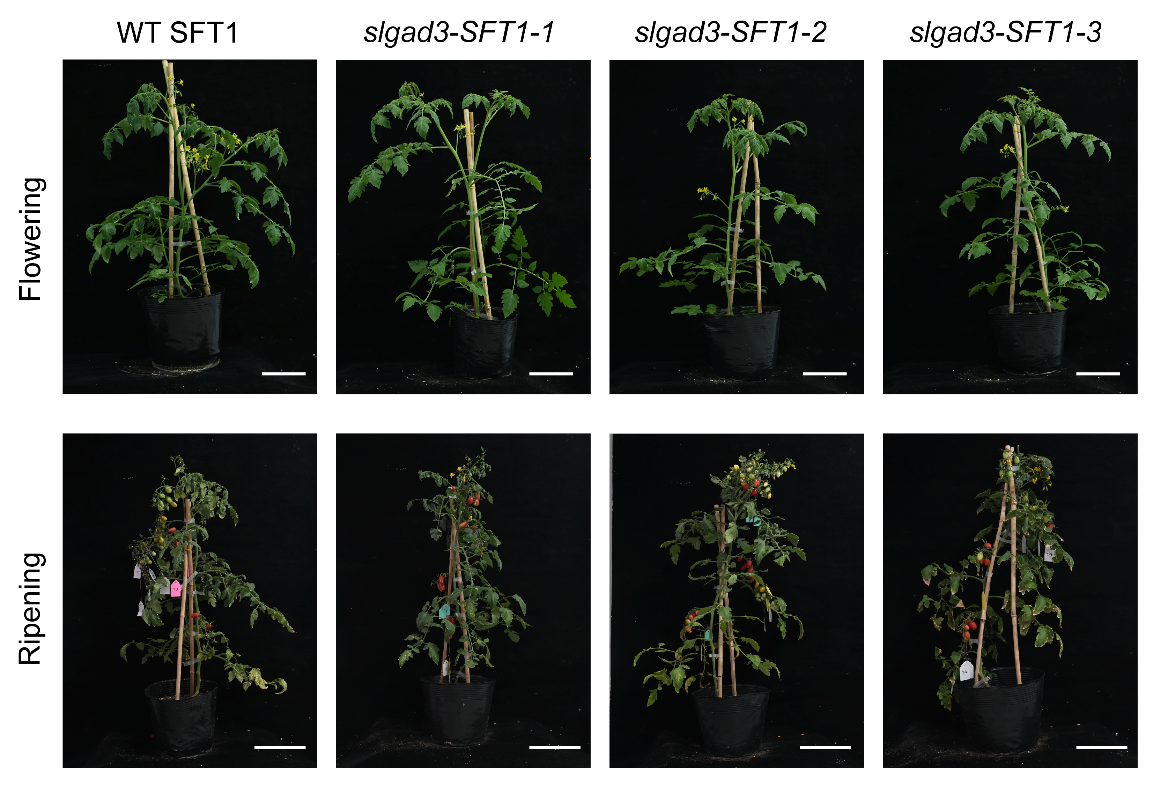
**

**Supplementary Fig 1.** The plant morphology of WT SFT1 and *slgad3-SFT1-1*, *slgad3-SFT1-2*, *slgad3-SFT1-3* mutant lines at flowering and fruit ripening stages. Scale bars = 10 cm.


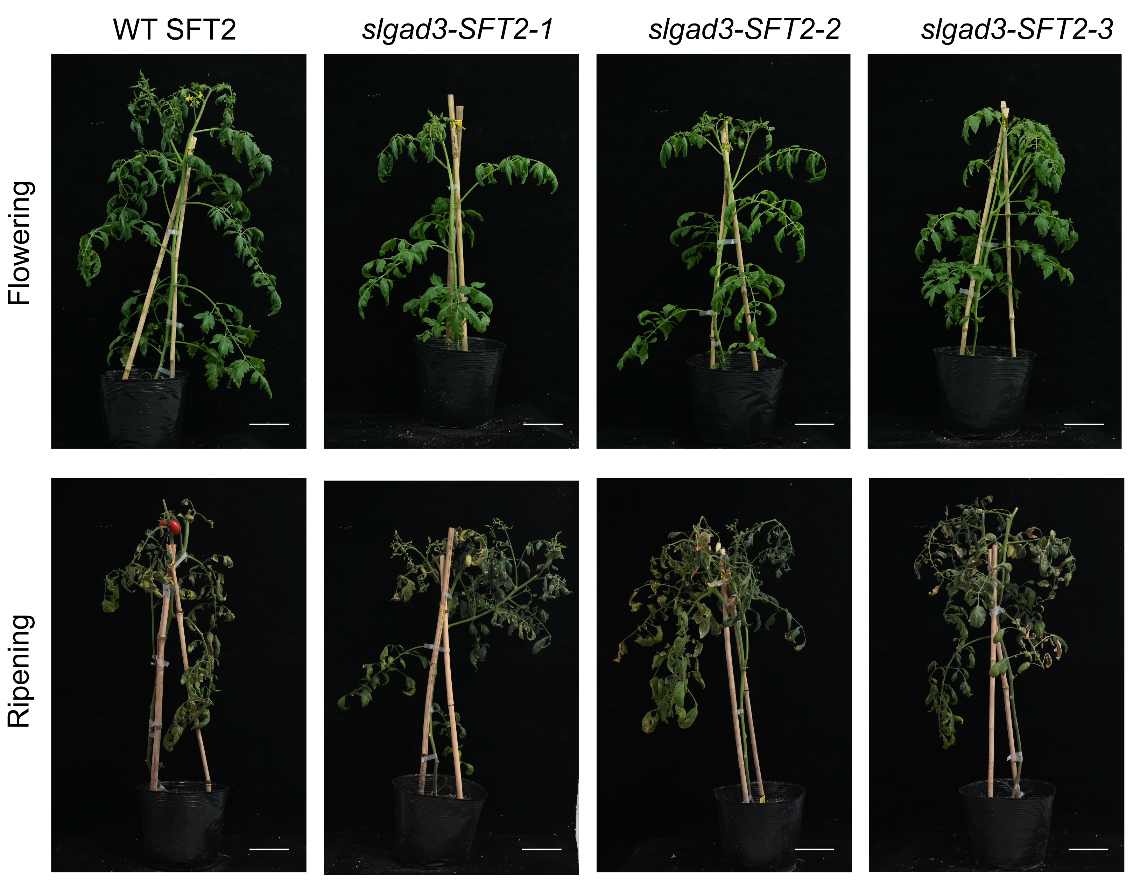


**Supplementary Fig 2.** The plant morphology of WT SFT2 and *slgad3-SFT2-1*, *slgad3-SFT2-2*, *slgad3-SFT2-3* mutant lines at flowering and fruit ripening stages. Scale bars = 10 cm.


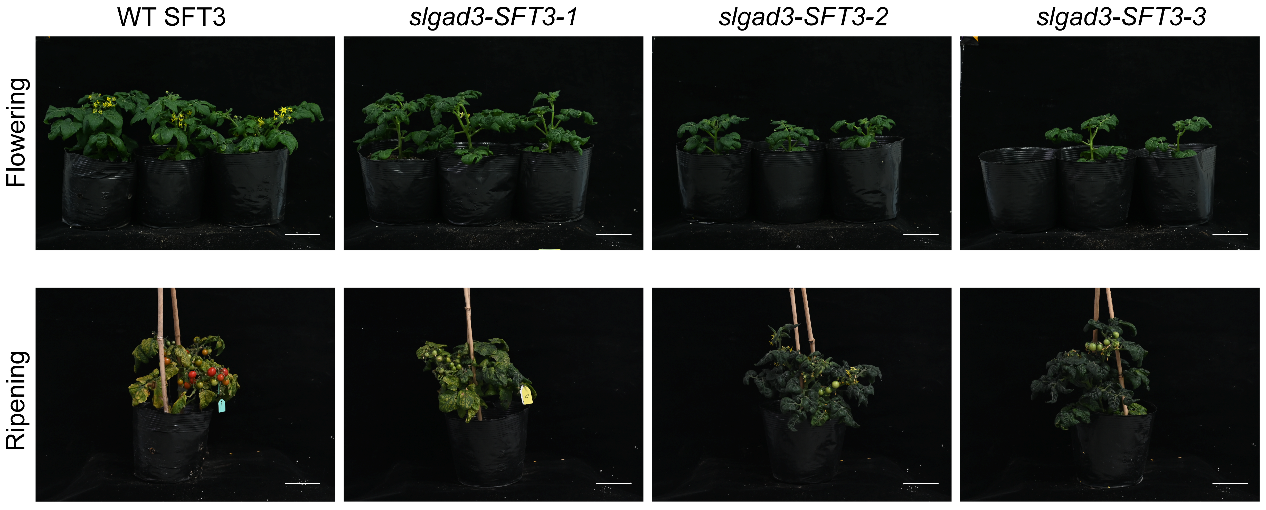


**Supplementary Fig 3.** The plant morphology of WT SFT3 and *slgad3-SFT3-1*, *slgad3-SFT3-2*, *slgad3-SFT3-3* mutant lines at flowering and fruit ripening stages. Scale bars = 10 cm.


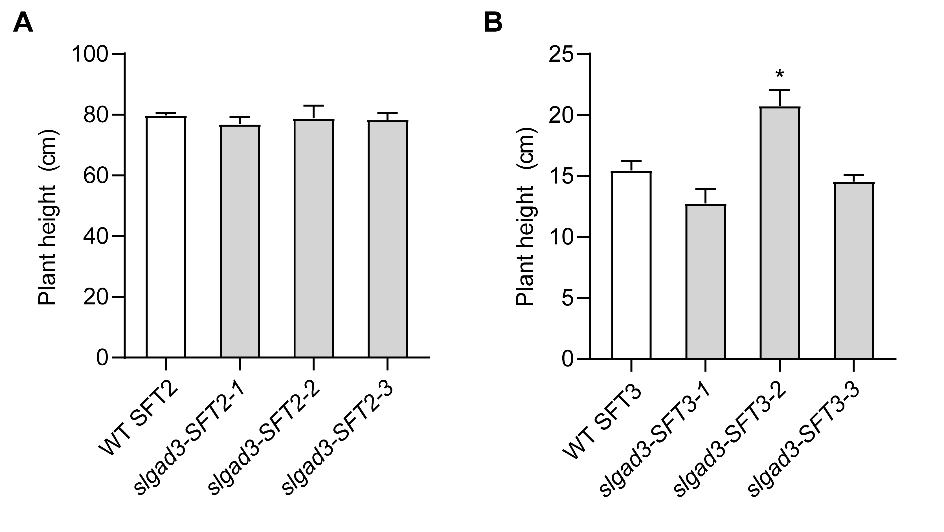


**Supplementary Fig 4.** The plant height of WT SFT2, SFT3 and corresponding mutant lines at ripening stage. Data were shown as mean ± SEM (n = 3); *P*-value calculated using Student's *t*-test and asterisks indicate significance level (*, P < 0.05; **, P < 0.01).


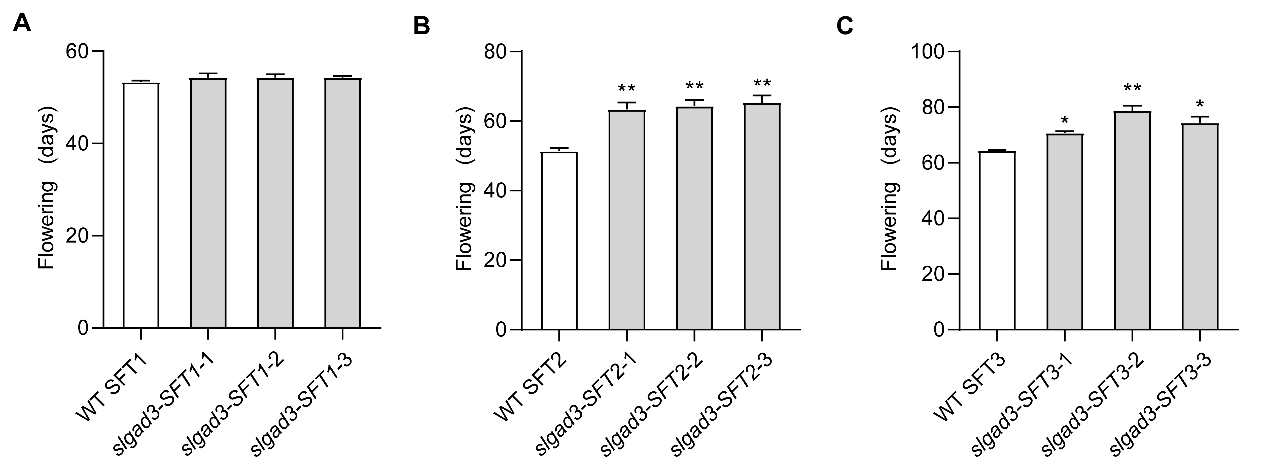


**Supplementary Fig 5.** The flowering time of WT SFT1, SFT2, and SFT3 and corresponding mutant lines. Data were shown as mean ± SEM (n = 3); *P*-value calculated using Student's *t*-test and asterisks indicate significance level (*, P < 0.05; **, P < 0.01).
